# NADPH Oxidase-Related Pathophysiology in Experimental Models of Stroke

**DOI:** 10.3390/ijms18102123

**Published:** 2017-10-11

**Authors:** Hiroshi Yao, Tetsuro Ago, Takanari Kitazono, Toru Nabika

**Affiliations:** 1 Laboratory of Neurochemistry, National Hospital Organization Hizen Psychiatric Center, Saga 842-0192, Japan; 2 Department of Medicine and Clinical Science, Graduate School of Medical Sciences, Kyushu University, Fukuoka 812-8582, Japan; agou@intmed2.med.kyushu-u.ac.jp (T.A.); kitazono@intmed2.med.kyushu-u.ac.jp (T.K.); 3 Department of Functional Pathology, Shimane University School of Medicine, Izumo 693-8501, Japan; nabika@med.shimane-u.ac.jp

**Keywords:** stroke, reactive oxygen species, focal ischemia, spontaneously hypertensive rats, pericytes, blood-brain barrier, ischemic penumbra

## Abstract

Several experimental studies have indicated that nicotinamide adenine dinucleotide phosphate (NADPH) oxidases (Nox) exert detrimental effects on ischemic brain tissue; *Nox*-knockout mice generally exhibit resistance to damage due to experimental stroke following middle cerebral artery occlusion (MCAO). Furthermore, our previous MCAO study indicated that infarct size and blood-brain barrier breakdown are enhanced in mice with pericyte-specific overexpression of Nox4, relative to levels observed in controls. However, it remains unclear whether Nox affects the stroke outcome directly by increasing oxidative stress at the site of ischemia, or indirectly by modifying physiological variables such as blood pressure or cerebral blood flow (CBF). Because of technical problems in the measurement of physiological variables and CBF, it is often difficult to address this issue in mouse models due to their small body size; in our previous study, we examined the effects of Nox activity on focal ischemic injury in a novel congenic rat strain: stroke-prone spontaneously hypertensive rats with loss-of-function in Nox. In this review, we summarize the current literature regarding the role of Nox in focal ischemic injury and discuss critical issues that should be considered when investigating Nox-related pathophysiology in animal models of stroke.

## 1. Introduction

The nicotinamide adenine dinucleotide phosphate (NADPH) oxidases (Nox)—which function solely to generate superoxide—are the predominant source of reactive oxygen species [1,2]. Nox-derived reactive oxygen species are thought to exert detrimental effects on the brain following a stroke. Paradoxically, tissue injury in stroke is caused by both the depletion (ischemia) and replenishment (reperfusion) of oxygen [2]. To date, seven members of the Nox family have been identified: Nox1-5, dual oxidase 1, and dual oxidase 2. Nox1-3, and Nox1-5 primarily produce superoxide, which is subsequently dismutated into H_2_O_2_, whereas Nox4, dual oxidase 1 and dual oxidase 2 directly produce H_2_O_2_ [1,2]. With the exception of Nox5, all Nox family members are multi-subunit enzymes; stabilization of Nox1-4 requires the assistance of the membrane-bound subunit p22*^phox^*. Expressions of Nox3 and 5 in the brain have only been documented in inner ear and brain cancer, respectively, and Nox5 is absent in rats and mice [1,2]. Furthermore, dual oxidase 1 and dual oxidase 2 are most commonly associated with thyroid function [1,2]. Hence, the present review discusses the impact of Nox1, Nox2, and Nox4 on stroke pathophysiology.

The physiological functions of Nox family members are extremely diverse, as these enzymes play key roles in host defense and inflammation, cellular signaling, gene expression, cellular death, cellular senescence, cell growth, oxygen sensing, angiogenesis, and various other biological processes [1]. Among Nox family members, Nox2 was first identified, and originally named gp91*^phox^* because of its high expression and activity in phagocytes; phox indicates phagocyte oxidase. A recent review by Sorce et al. summarized the physiological and pathological roles of Nox in the central nervous system (CNS) [3]. Nox2 and Nox4 are the main Nox isoforms expressed in the CNS. Under physiological conditions, most Nox isoforms are expressed only to a negligible extent in the CNS. In the CNS, Nox2 is expressed mostly in microglia. High levels of Nox2 activity was associated with decreased survival in amyotrophic lateral sclerosis patients and autoimmune demyelination [3]. Nox2 activity in blood samples may be a useful biomarker for neuroinflammation that is associated with neurodegenerative diseases.

Research has demonstrated that Nox is also involved in the physiological functions of the vasculature as well as hypertension [1,4], the latter of which represents a major risk factor for stroke. Superoxide rapidly interacts with nitric oxide to form peroxynitrite, resulting in vasoconstriction and increased blood pressure [4]. Basal and stimulated generation of superoxide from Nox is one to two orders of magnitude higher in intracranial cerebral arteries than in systemic arteries, and activation of Nox in cerebral arteries can cause vasodilation under normal conditions [5]. In addition, Nox4 may function as the major catalytic component of an endothelial NADPH oxidase [6]. Previous studies have reported that rat basilar artery endothelial cells exhibit abundant expression of Nox1, while Nox1 expression in aortic endothelial cells is marginal, indicating that different vascular beds may contain different sets of Nox proteins [7]. Several studies have indicated that Nox4 activity is increased in cerebral arteries during chronic hypertension, and that this increase in expression is associated with greater production of superoxide and vasodilatation in spontaneously hypertensive rats [8].

Stroke has a secondary injury component following a primary ischemic event; secondary injury involves a cascade of injury pathways that exacerbate tissue damage including inflammation, excitotoxicity, oxidative stress, loss of ion homeostasis, and increased blood-brain barrier (BBB) permeability [9]. Neurally-elicited inflammatory process (i.e., neurogenic inflammation) plays a key role in the secondary injury cascades (e.g., enhanced permeability of the BBB after stroke) that evolve following a stroke. Excessive Nox-derived reactive oxygen species likely lead to neurodegeneration via breakdown of the BBB, and/or via neuronal apoptosis [10]. However, whether the effects of Nox on stroke outcomes are due to direct increases in oxidative stress at the site of ischemia or indirect increases due to the modification of physiological variables (e.g., blood pressure or cerebral blood flow (CBF)) remains unknown. Although the mechanisms underlying ischemia/reperfusion injury caused by Nox-related oxidative stress remain to be fully elucidated, Nox are promising therapeutic targets for the treatment of stroke.

**Figure 1:**
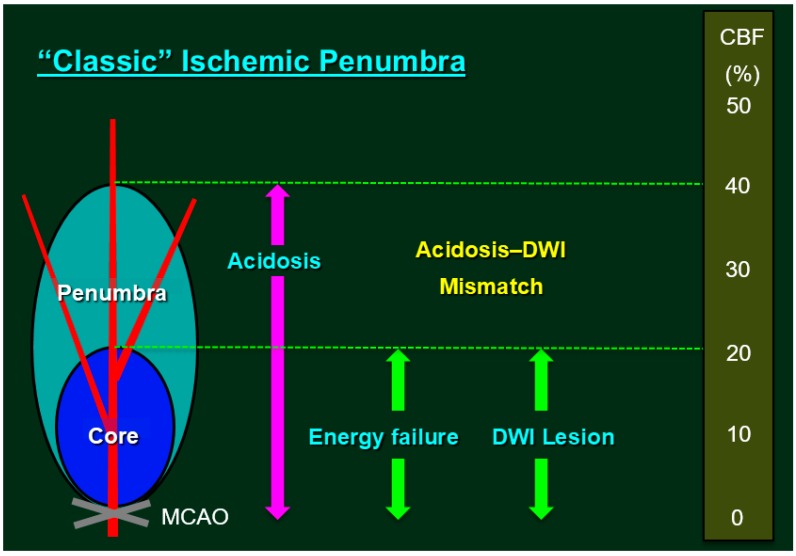
Classic Ischemic Penumbra. The ischemic penumbra is classically defined as an ischemic brain region with cerebral blood flow (CBF) between the upper threshold of electrical silence and the lower threshold of energy and ion pump failure [11,12]; the green dashed lines indicate the upper and the lower thresholds. Regions exhibiting CBF in the ischemic core range (CBF <20% of control values) had a 96% probability of undergoing infarction, while zones with higher CBF (>40% of control) were largely spared from infarction [13]. Under experimental conditions, the most reliable method for localizing the ischemic core involves inducing a loss of adenosine triphosphate (ATP), although tissue acidosis can be used as a biochemical marker of the ischemic core plus the penumbra [14]. Research has demonstrated that pH-weighted magnetic resonance imaging and diffusion-weighted imaging (DWI) mismatch (i.e., cytotoxic edema due to ATP depletion) can provide a more comprehensive zone of penumbra [15]. In routine clinical settings, however, lesions detected via early DWI can be used to define the ischemic core, while the adjacent moderately ischemic brain tissue can be identified as the penumbra via perfusion-weighted imaging (i.e., perfusion-weighted imaging/DWI mismatch as a surrogate marker for penumbra) [16,17]. Previous studies have demonstrated that, even after early reperfusion with tissue plasminogen activator, sustained reversal of diffusion abnormalities was minimal, indicating that the infarct core is well represented by the acute DWI lesion [18].

In this review, we present a brief overview of experimental stroke models and pathophysiology associated with the “classic” concept of the ischemic penumbra. The ischemic penumbra is classically defined as an ischemic brain region with CBF between the upper threshold of electrical silence and the lower threshold of energy and ion pump failure (see legends for Figure 1). We highlight findings obtained in *Nox* knockout mouse models of experimental stroke, as well as the effects of Nox on cerebral blood vessels and the BBB, focusing on the role of pericytes. In addition, we discuss several confounding factors associated with experimental models of stroke based on our recent findings in stroke-prone spontaneously hypertensive rats (SHRSP), which exhibit Nox dysfunction.

## 2. Experimental Models of Stroke

Models of acute brain infarction associated with the occlusion of major cerebral arteries are developed by inducing focal ischemia or middle cerebral artery occlusion (MCAO) in animals [19]. Here, we summarize the characteristics of the most commonly utilized rat and mouse models of stroke (Table 1).

### 2.1. Distal Middle Cerebral Artery Occlusion

The pathophysiological consequences of distal MCAO distal to the lenticulostriate branches have been investigated in 35–40 day old Wistar Kyoto rats (WKY) and SHRSP. Such studies have revealed that, even in young SHRSP with mild hypertension, distal MCAO lesions are larger than those observed in normotensive WKY [20].

### 2.2. Proximal MCAO

Tamura et al. first described methods for MCAO proximal to the lenticulostriate arteries, which produces infarction of both the cortex and the lateral portion of the striatum [21]. Duverger and MacKenzie subsequently modified this method using SHRSP, SHR, and three normotensive strains (WKY, Sprague-Dawley rats, and Fischer-344 rats) [22]. Proximal MCAO results in a considerably larger infarction in hypertensive rats than in normotensive rats. Moreover, minimal variability was observed among the hypertensive strains [coefficient of variation (C.V.) = 7–8%] when compared with findings in the three normotensive strains (C.V. = 20–49%).

### 2.3. Intraluminal Suture Occlusion

The intraluminal suture model, developed by Koizumi et al. [23] and Longa et al. [24], is undoubtedly the most frequently used model of focal ischemia in rats and mice [25]. This method is advantageous in that it is easy to perform, minimally invasive, and—most importantly—does not require craniectomy. Although reperfusion of ischemic brain tissue is critical for restoring normal function, it can paradoxically result in secondary damage or reperfusion injury. Several lines of evidence have suggested that post-ischemic oxidative stress and inflammation contribute to brain injury [26]. As reperfusion injury is the condition of ischemia aggravated by the occurrence of reperfusion more so than in the case where reperfusion does not occur (i.e., permanent occlusion), in order to see reperfusion injury it is necessary to make a comparison with permanent occlusion. Furthermore, from a critical point of view, the intraluminal suture model is a top of the internal carotid artery occlusion model rather than a MCAO model [27]. Consequently, this model exhibits a wide zone of ischemia, and because the mortality rate is high in the case of permanent occlusion, the permanent occlusion group and the reperfusion group—that shared the same time lapse—cannot be compared.

### 2.4. Photothrombotic MCAO in Spontaneously Hypertensive Rats

Several studies have indicated that the effects of thrombotic stroke on compromised brain tissue may differ from those due to cerebral ischemia induced by mechanical occlusion of intracranial or extracranial brain arteries [28,29]. Prado et al. first used SHR as a model of a photothrombotic distal MCAO model without common carotid artery occlusion [30]. In our experience, the photothrombotic distal MCAO model in SHR yields a highly reproducible infarct volume (average C.V. = 21%) and does not entail extensive surgery or opening of the dura, thereby avoiding unacceptable local tissue trauma at the site of MCAO [31,32,33]. This model encompasses appropriate physiological monitoring, associated risk factors for stroke, and clinically relevant pathophysiology of thrombosis. Watson et al. achieved successful ultraviolet laser-induced reperfusion in animal models of photothrombotic stroke [34], which has also been applied in SHR [31,32].

### 2.5. Ischemic Penumbra

The term “penumbra” refers to the salvageable tissue surrounding the ischemic core (Figure 1). Focal ischemia essentially consists of an ischemic core and penumbra, although it remains unclear which model of focal ischemia is most relevant for investigating phenomena in the penumbra. Proximal MCAO produces two ischemic vascular territories associated with the lenticulostriate arteries and cortical branches of the MCA, the former of which are end-arteries, and therefore treatment-resistant (i.e., ischemic core without collateral perfusion). The intraluminal suture model involves internal carotid artery occlusion rather than pure MCAO [27]. In clinical settings, the arterial obstruction site strongly predicts infarct growth and clinical outcome; internal carotid artery occlusion has been associated with uniformly poor prognosis and poor response to tissue plasminogen activator therapy [35]. Although the infarcts produced by distal MCAO in normotensive rats are small with a presumably limited size of penumbra, distal MCAO alone—occlusion of one major cerebral artery with collateral perfusion—in SHR results in substantial infarct size and an acceptable amount of penumbra [31,32].

Taken together, these findings indicate that distal MCAO in SHR represents the best choice for an experimental model of human stroke. In our experience with distal MCAO in SHR, early restoration of perfusion confers advantages on the ischemic brain; our findings indicated that early reperfusion attenuated damage in the cortical region, which was regarded as the penumbral zone [36]. Although reperfusion after a relatively long period of ischemia did not decrease final infarct size or fodrin breakdown, as an index of evolving ischemic injury was attenuated in the penumbra [37]. These results support the notion that distal MCAO in SHR provides a rational approach for investigation phenomena associated with the ischemic penumbra.

### 2.6. Nox in Experimental Models of Stroke

Because neither Nox-specific nor isoform-specific inhibitors for NADPH oxidases are not available, Nox knock-out mice are widely utilized to assess the effects of Nox on stroke pathophysiology (Table 2).

### 2.7. Nox2

Among the Nox isoforms, Nox2 has been the most widely studied in the context of focal ischemia or stroke. Transient focal ischemia in *Nox2* knock-out mice results in smaller infarct volume than that induced in wild-type mice, suggesting that superoxide generated from NADPH oxidase plays a major role in mediating ischemia-reperfusion injury [38,39,40,41]. Apocynin is currently the most selective inhibitor of the Nox family and is thought to inhibit activation of the enzyme by preventing translocation of p47*^phox^*. In several earlier studies, both Nox2 depletion and apocynin treatment significantly reduced infarct volume compared to that of untreated wild-type mice [39,40]. Furthermore, pre-treatment with apocynin also reduced neurological impairments and mortality in wild-type but not *Nox2* knockout mice. The authors further reported that pre-treatment with apocynin significantly reduced superoxide production as detected using in situ dihydroethidium methods following ischemia-reperfusion [41]. However, *Nox2* knockouts failed to attenuate infarct size in the absence of reperfusion (i.e., permanent occlusion) [42]. Additional studies indicated that the larger infarct region observed in male mice when compared with female mice after transient occlusion (but not permanent suture occlusion) was dependent on Nox2 [43].

Oxidative stress induced by ischemia-reperfusion causes endothelial dysfunction via increased expression and activity of Nox2 in the cerebral artery. This endothelial dysfunction appears to be due to impaired NO-mediated vasodilation most likely via the direct inactivation of NO and the formation of peroxynitrite. A study reported that *Nox2* knockout decreased superoxide production in the MCA, attenuated *N*^ω^-Nitro-L-arginine methyl ester-induced vasoconstriction, and reduced infarct size compared with the wild-type mice [44]. In accordance with the effects of Nox2 on vascular function, genetic depletion of Nox2 attenuates suture occlusion-induced infarction and early BBB dysfunction [45]. Nox2 knockout showed transient nature of protection (i.e., reduced infarct volume at 24 h, but not at 6 or 72 h post-stroke), suggesting that other factors mediate infarct size in acute stroke. Furthermore, Nox2 deletion increased re-vascularization of the damaged brain by 72 h [46]. Taken together, these findings indicate that *Nox2* knockout generally exerts protective effects against focal ischemia-reperfusion but not against permanent MCAO.

### 2.8. Nox1

Angiotensin II-stimulated increases in superoxide generation in intact cerebral arteries were threefold higher in wild-type than in *Nox1* knockout mice; although cortical (i.e., penumbra) infarct volume was fourfold greater in Nox1 knockout mice than in wild-type mice, no such effects were observed on subcortical infarct volume, suggesting that Nox1 exerts protective effects against focal ischemic injury [47]. After 1 h of ischemia and 23 h of reperfusion, infarct volume was reduced by 44% in *Nox1* knock-out mice relative to levels observed in wild-type mice; however, no difference in infarct size was observed between *Nox1* knockout and wild-type mice with permanent occlusion (2 h of occlusion time) [48]. Both infarct size and neuronal death after 90 min of suture occlusion in male Wistar rats were significantly reduced by adeno-associated virus-mediated transduction of *Nox1* short hairpin RNA (i.e., *Nox1* knockdown) [49].

### 2.9. Nox4

*Nox4* knockout—but not *Nox1* or *Nox2* knockout—exerted protective effects against both transient and permanent focal ischemia; mice deficient in Nox4 were also protected from oxidative stress, BBB disruption, and neuronal apoptosis [50]. Surprisingly, in this study, photothrombosis-induced, not photothrombotic MCAO-induced, cortical infarction was attenuated in *Nox4* knock-out mice when compared to wild-type mice. Nox4 is highly expressed in the endothelium and contributes to hydrogen peroxide (H_2_O_2_) formation [1,2,4]. Although H_2_O_2_—the dismutation product of O_2_^−^—elicits adverse effects on the vascular system (e.g., smooth muscle cell hypertrophy, metalloproteinase activation, and NOS inhibition, etc.), Nox4-derived H_2_O_2_ also induces positive endothelial effects. For example, Nox4 depletion attenuates ischemia-induced angiogenesis (femoral artery ligation) and exacerbates angiotensin II-induced vascular dysfunction [51].

### 2.10. Pericyte Nox4 in Focal Brain Ischemia

Pericytes have been recognized as a key component of the BBB or neurovascular unit [52]. Although endothelial cells primarily contribute to the formation of the BBB by expressing tight junction-related proteins (e.g., claudins and occludins) on their plasma membranes, high coverage ratios of pericytes to endothelial cells and the interaction between the two cell types are indispensable for maintaining the BBB [53]. In addition, the pericytes localized in pre-capillary arterioles may be responsible for the neuronal activation-coupled vasodilatation that increases CBF (i.e., neurovascular coupling) [54]. Therefore, dysfunction or functional loss of pericytes can directly disrupt the BBB and impair CBF regulation, thereby leading to neuronal dysfunction or death.

Among the Nox family proteins, cultured brain pericytes specifically express Nox4 [55]. Hypoxia is a significant up-regulator of Nox4 in the pericytes, as well as in other types of cells [55,56]. Several studies have reported a close association between hypoxia-inducible factor-1 and Nox4, suggesting that Nox4 functions at multiple sites, including both upstream and downstream of hypoxia-inducible factor-1 [57,58]. Another important target of Nox4 may be nuclear factor kappa B (NFκB). Previous studies have indicated that Nox4 overexpression leads to the activation of IκB kinase (IKK), which in turn induces the degradation of IκB and the subsequent stabilization and phosphorylation of NFκB in cultured brain pericytes [59]. Because NFκB is a pro-inflammatory transcription factor, microvascular cells with upregulated Nox4 expression may produce pro-inflammatory cytokines/chemokines, thereby enhancing inflammation. However, NFκB has also been observed to enhance the survival of microvascular cells under hypoxic conditions [60]. Furthermore, Nox4 overexpression enhances the proliferation of cultured pericytes, while *Nox4* knockdown attenuates such proliferation [55].

Nox4 is expressed ubiquitously in various cell types; however, immunohistochemistry experiments have demonstrated that the levels of Nox4 expression in the brain are very low under physiological conditions [50,59]. Indeed, Nox4 was originally identified as one of the Nox isoforms specific for the kidney, which senses anemia and systemic hypoxia to produce erythropoietin under physiological conditions [61]. However, the expression of Nox4 is markedly upregulated in microvascular cells (including pericytes), particularly in peri-infarct areas, with peak expression occurring approximately 3–5 days after the onset of both permanent and transient MCAO [59]. Interestingly, the extent of Nox4 upregulation was greater in permanent than in transient MCAO with 1–2 h of ischemia [59], which may support the notion that Nox4 is upregulated in response to hypoxia/ischemia [56].

As mentioned above, research has demonstrated that infarct volumes were smaller and accompanied by decreased BBB leakage and neuronal apoptosis in systemic *Nox4* knockout mice than in wild-type mice after both permanent and transient MCAO [50]. In this study, *Nox4* deletion had a greater impact on phenotypic changes (i.e., reduction of neuronal damages after MCAO) than deletion of *Nox1* or *Nox2* in mice [50]. Furthermore, we demonstrated that infarct volume was significantly larger with enhanced BBB breakdown in mice with pericyte-specific overexpression of Nox4 (Tg-Nox4) than in their littermate controls in a permanent MCAO model [59]. These findings indicate that upregulation of Nox4 in microvascular pericytes exerts a detrimental effect in the acute phase of ischemic stroke. This study reported that the activation of NFκB in the ischemic hemisphere following permanent MCAO was significantly greater in Tg-Nox4 mice than in wild-type controls, as predicted by in vitro experiments, and enzymatic activity of matrix metalloproteinase-9 (MMP-9)—known to be involved in the breakdown of the BBB—was significantly greater and prolonged in Tg-Nox4 [59]. Thus, Nox4-mediated increases in the activation of NFκB may account in part for the phenotypic changes observed in Tg-Nox4 mice. In addition, previous researchers have suggested that reactive oxygen species may cause contraction of pericytes, thereby impairing capillary reperfusion after ischemia and aggravating brain ischemia [62]. It is also possible that Nox4 in pericytes is associated with ROS-induced capillary constriction during ischemia-reperfusion.

MMP-9 activation occurs biphasically following acute brain ischemia, while immediate MMP-9 activation occurs within one day after ischemic stroke due to the infiltration of inflammatory cells, and delayed activation is derived from vascular cells in the brain, contributing to angiogenesis and subsequent tissue repair [63]. Based on the profile of Nox4 expression in the ischemic brain [59], pericyte-Nox4 may participate in the latter process. For example, in a hindlimb ischemia model, Nox4 overexpression in endothelial cells promoted the angiogenesis and beneficial restoration of blood flow in the chronic phase [64]. Poststroke fibrotic changes within infarct areas occurring sequentially after angiogenesis may play important roles in the tissue repair process leading to functional recovery [53,54,55,56,57,58,59,60,61,62,63,64,65,66,67]. The involvement of Nox4 in fibrotic responses has been demonstrated in some diseases out of central nervous system [68]. Thus, there is a possibility that microvascular Nox4 may promote angiogenic and/or fibrotic responses in a chronic phase even in brain ischemia. In this context, the effects of Nox4 may be similar to those of vascular endothelial growth factor (VEGF) during brain ischemia, although VEGF is established as an angiogenic and neuroprotective molecule in the brain, it appeared to function detrimentally in the very acute phases of brain ischemia due to enhancement of BBB breakdown [69]. These findings indicate that the effects of Nox4 may vary in different organs or tissues and according to the type of injury or timing of disease states.

### 2.11. Stroke-Prone Spontaneously Hypertensive Rats (SHRSP) with Loss-of-Function in Nox

As mentioned above, although knockout of Nox attenuated focal ischemic injury in most but not all studies, the mechanisms by which Nox attenuates ischemic injury are not clear. Nox may exert direct effects on stroke outcome by increasing oxidative stress at the site of ischemia, or indirect effects by modifying physiological variables such as blood pressure and/or cerebral blood flow (CBF) [19]. In particular, blood pressure levels and antihypertensive therapy critically affects CBF via collateral and infarct size in hypertensive rats [70,71]. Because measurement of physiological variables and CBF in mice is often difficult due to their small body size, we attempted to construct a new rat model lacking Nox activities. The Matsumoto Eosinophilia Shinshu (MES) rat was found to harbor a deletion of four nucleotides in Intron 4 of the cytochrome b-245, alpha polypeptide (*Cyba*) gene, which resulted in an abnormal splicing [72]. As *Cyba* encodes p22*^phox^*, this strain lacked expression of the p22*^phox^* protein and showed loss of Nox activities. By introgressing the mutated *Cyba* gene from the MES strain, we constructed a congenic strain (referred to as SP.MES hereafter). Because Nox1-4 need to form heterodimers with the membrane-bound p22*^phox^* subunit [1], SP.MES lacks Nox activities on the background of the SHRSP. Although we have reported the results of photothrombotic distal MCAO in this new congenic strain [73], the findings are summarized below.

Resting MABP in SP.MES decreased slightly but significantly compared with that in the control PM0/SHRSP. In our previous study, CBF decreased to 37 ± 13% in SP.MES and 35 ± 17% in PM0/SHRSP at 10 min after MCA occlusion. The changes in CBF from 10 to 60 min after MCA occlusion were 7 ± 8% and −4 ± 8% in SP.MES and PM0/SHRSP, respectively (*p* = 0.006). The distal MCA pattern was more complex in SP.MES when compared to PM0/SHRSP. Although infarct volume in SP.MES was not significantly different from that in the PM0/SHRSP (89 ± 39 mm^3^ vs. 83 ± 35 mm^3^, respectively), we had revealed that the infarct volume with complex MCA had been larger than that in simple MCA [33]. We then adjusted infarct volume for the branching pattern as a covariate in an analysis of covariance; the adjusted mean of infarct volume was significantly smaller in SP.MES than in PM0/SHRSP (67 (95% CI 46 to 87) mm^3^ vs. 100 (95% CI 82 to 118) mm^3^, *p* = 0.032).

**Figure 2:**
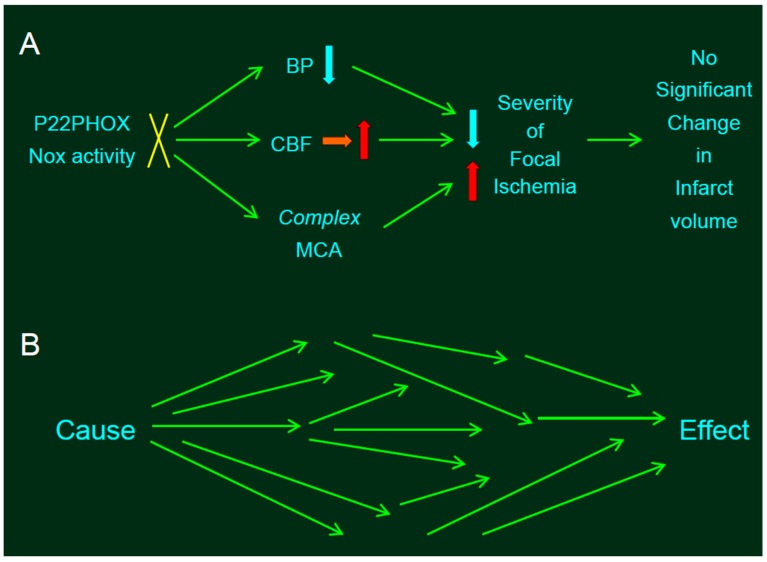
Statistical problems in experiments involving genetic manipulation. (**A**) The absence of p22*^phox^* protein did not affect infarct size produced by distal middle cerebral artery occlusion (MCAO). Resting mean blood pressure (MABP) in SP.MES rats was decreased slightly but significantly relative to that observed in control PM0/SHRSP rats, along with mitigated CBF. Thus, slightly but significantly decreased resting blood pressure (BP)—along with mitigated CBF—would decrease the infarct size in SP.MES, whereas more complex MCA might have resulted in larger infarction. The blue, red, and orange arrows indicate decrease, increase, and no significant change, respectively. (**B**) Such divergent effects of genetic manipulation provoke a critical problem. If the experiments were adequately randomized, we can conclude that the effect—significantly different between the groups—was due to a single cause, because the background factors between the groups were same; in other words, no factor except for a single cause can be the reason for the difference between the groups (i.e., the single effect) after randomization. However, when a single cause (e.g., knockout of a gene) induced multiple influences converging into a single effect (e.g., infarct volume), this assumption is invalid.

Functional loss of Nox activity associated with the absence of the p22*^phox^* protein did not mitigate the size of infarction produced by distal MCA occlusion as mentioned above. We speculated that decreased superoxide production and lower resting blood pressure would decrease the infarct size in SP.MES, whereas more complex MCA—as observed in SP.MES—would result in larger infarction. These divergent phenotypic effects caused by Nox dysfunction may explain the similarity in infarct size between SP.MES and PM0/SHRSP. Such divergence represents a major limitation of knockout strategies in stroke research (Figure 2).

### 2.12. Nox and Branching Morphogenesis

The distal MCA is considered to be more complicated when the number of classification of the distal MCA pattern increases from Type I to Type VII (i.e., more branches accompanied by tortuosity) [33]. In our previous study, we observed that distal MCA patterns were more complex in SP.MES (median = 3, interquartile range (IQR) = 3–5) than in PM0/SHRSP (median = 2, IQR = 1–3), indicating that oxidative stress may alter complex MCA into simple MCA. In a retrospective analysis of the distal MCA, simple patterns were more prevalent in SHRSP, while more complicated patterns were observed in WKY; SHR exhibited intermediate features relative to those observed between SHRSP and WKY. Because SHR were initially obtained by selective inbreeding of WKY with the highest blood pressure, and SHRSP were established from the A substrain of the SHR, these three substrains are genetically very close [19]. Nonetheless, distal MCA patterns among these three substrains were considerably different.

The vascular system is probably the most prominent instance of hierarchical tubular network characterized by a single topological feature (i.e., branching) [74,75]. To our knowledge, a direct involvement of reactive oxygen species or of the Nox activity has not been suggested in the context of branching morphogenesis of blood vessels. Reactive oxygen species exert profound effects on vascular smooth muscle cell growth and migration. Earlier studies have indicated that angiotensin II infusion significantly increased aortic hypertrophy in transgenic mice overexpressing Nox1 when compared with wild-type littermates [76]. Nox4-derived reactive oxygen species have also proven critical to the maintenance of the differentiated phenotype of vascular smooth muscle cells [77]. A theory—age-related loss of complexity—was proposed based on nonlinear analyses [i.e., fractal-like (self-similar) branching architecture] [78]. Interestingly, decreased branching complexity of retinal vessels was associated with increasing age and lacunar infarction in stroke patients, which suggested that more pathological status brought less complex blood vessels or loss of complexity [79]. SHRSP dominantly showed “loss of complexity” of MCA compared with its normotensive counterpart (WKY). Since it is widely believed that SHRSP had a higher level of the oxidative stress [80], the hypothesis seems intuitively attractive that increased levels of oxidative stress in SHRSP induced the simple pattern of MCA.

### 2.13. Effects of Nox on Blood Pressure

Under physiological conditions, superoxide and hydrogen peroxide produced by Nox participate in cardiovascular regulation [81]. Physiological variables such as blood pressure are critically important in experimental stroke; for example, congenic removal of a blood pressure quantitative trait locus (decreased blood pressure by 12% or −29 mmHg) attenuated infarct size produced by MCAO [70]. Our previous study mentioned above showed slightly but significantly decreased blood pressure in SP.MES (i.e., Nox dysfunction due to the absence of p22*^phox^* protein) when compared with SHRSP/PM0, suggesting that decreased levels of resting or pre-stroke blood pressure in hypertensive rats would attenuate infarct size produced by MCAO [73]. Therefore, decreases in blood pressure due to the absence of p22*^phox^* may explain at least in part the protective effects against focal ischemic injury observed in SP.MES. Nox is an important sources of reactive oxygen species in blood pressure regulation. Although Nox1 was involved in the late phase of the response to angiotensin II (i.e., after seven days of continuous infusion of angiotensin II), no significant difference in baseline blood pressure was observed between *Nox1* knockout and wild-type mice [82,83]. While one study reported that baseline blood pressure was lower in *Nox2* knockout mice than in wild-type mice [84], another study reported that Nox2 deficiency did not protect against the development of hypertension in another study [85]. Nox2 is expected to impair NO-mediated relaxation and lead to vascular contraction, while endothelial Nox4 enhances vasodilatation and reduce blood pressure [86]. These results highlight the complexities regarding the interactions between Nox activity and blood pressure regulation. Furthermore, one of the authors’ group reported that blood pressure response to glutamate microinjection into the rostral ventrolateral medulla was significantly greater in SHRSP than in SP.MES, SHR and WKY, suggesting that higher oxidative stress or Nox activity in the regulatory center of the sympathetic nerve activity in SHRSP [87].

## 3. Perspectives—Nox Knockout in Rats

As four NOX subtypes rely on p22*^phox^* to maintain their activity, interpretation of the observations on SP.MES described above is not straightforward. In this context, it is of interest to study phenotypes of SHRSP depleted with each Nox subtype. Recently, a groundbreaking method, genome editing using CRISPR/CAS9, has been introduced to modify genomes in any types of animals [88].

This method has many advantages such as high efficiency, low cost, and is less time-consuming. In addition, the most important feature for us is that it can be applied to any strains of rats, particularly genetic model rats for human diseases such as SHRSP.

To address the questions raised in above sections, we planned to construct SHRSP-depleted Nox subtypes using the CRISPR/CAS9 method. We have successfully established knockout lines each for *Nox2* and *Nox4*, respectively. Preliminary results suggested that *Nox2* and *Nox4* knockout decreased and increased baseline blood pressure in SHRSP, respectively. We will employ these new knockout rats in MCAO experiments to reveal the roles of different Nox subtypes in the development of cerebral infarction.

## 4. Concluding Comments

Although distal MCAO in SHR is considered to be the best choice for an experimental model of human stroke, the suture model using knockout mice are widely utilized to assess the effects of Nox on stroke pathophysiology. However, due to the technical problems in the measurement of physiological variables or CBF in mice with their small body size, it remains unclear whether Nox affects the stroke outcome via direct increases in oxidative stress at the site of ischemia, or via indirect effects by modifying physiological variables such as blood pressure or CBF. Nonetheless, Nox knockout generally conferred protection against focal experimental stroke, suggesting that Nox exerts detrimental effects of Nox on stroke outcome. Notably, the pericyte-specific overexpression of Nox4 played a detrimental role in the acute phase of ischemic stroke by increasing the size of the infarct area and enhancing breakdown of the BBB.

To date, evidence further suggests that the effects of Nox isoforms on focal ischemic injury vary according to cell type (neuron, glia, microglia, blood vessels, or pericytes), the presence of ischemia with or without reperfusion, and disease state (acute or chronic). The absence of p22*^phox^* resulted in divergent effects (presumably beneficial vs. detrimental phenomena), representing a substantial limitation in experimental stroke research. As such, knockout strategies cannot provide a simple answer regarding whether a specific gene or protein is beneficial or detrimental for stroke. SHRSP with a higher level of the oxidative stress showed simple MCA pattern or “loss of complexity” of MCA compared with its normotensive counterpart. Based on this finding, we propose that increased levels of oxidative stress induce decreases in the complexity of the MCA.

Because hypertension is one of the major risk factors for stroke, the Nox-related mechanisms involved in blood pressure control are of great interest. Such Nox-related mechanisms in vascular morphogenesis and blood pressure control should be further examined in Nox isoform-specific “knockout rat” in SHRSP.

In conclusion, this review indicates that Nox is a potential therapeutic target in acute ischemic stroke. Furthermore, inhibition of Nox activity may play a role of stroke prevention through blood pressure lowering or attenuated vascular pathophysiology. The mechanisms by which Nox exert protection against stroke should be further elucidated.

## Figures and Tables

**Table 1:** Experimental models of stroke.

Models	Anatomic Sites of MCAO	Duration	Characteristics	Disadvantage
Distal MCAO	MCAO distal to rhinal fissure	P	Distal MCAO was studied for the first time in SHRSP, revealing increased stroke sensitivity by hypertension.	The infarct size and penumbra are too small to evaluate the effects of pharmacotherapeutic agents in normotensive rats.
Proximal MCAO	MCAO proximal to lenticulostriate arteries	P (T)	The subtemporal approach method have emerged as a standard method of proximal MCAO.	The procedure is surgically demanding and may induce local traumatic effects.
Intraluminal suture occlusion	MCA origin and the proximal segment of ACA	T (P)	The procedure is easy to perform, minimally invasive, and does not require craniectomy.	This model has a wide ischemic zone, and the mortality rate is high in the case of PO.
Photothrombotic MCAO	Photochemical MCAO distal to rhinal fissure	P T	Photothrombotic MCAO in SHR yields a highly reproducible infarct volume, and does not requireopening of the dura.	Same as mentioned for distal MCAO.

MCAO, middle cerebral artery occlusion; MCA, middle cerebral artery; ACA, anterior cerebral artery; P, permanent occlusion; T, transient occlusion; SHRSP, stroke-prone spontaneously hypertensive rats.

**Table 2:** Effects of *Nox* knock-outs on experimental stroke.

Author Ref.	Year	Mice (WT: C57 Bl/6J)		T/P	BP	CBF	Nox Isoform	Outcome	Protection by KO
		Age	Sex						
Walder [36]	1997	8–10 wk	m	T	NA	NA	Nox2	Infarct volume was reduced by 46% in KO mice compared with WT mice.	Yes
Kahles [43]	2007	7–9 wk	m	T	NA	NA	Nox2	BBB disruption and lesion volume were largely attenuated in KO mice.	Yes
Chen [37]	2009	NA	m	T	NA	NS	Nox2	Mean infarct volume was 106.2 mm3 in WT mice, and 52.0 mm^3^ in KO mice.	Yes
Jackman [39]	2009	5–9 wk WT 8–12 wk KO	m m	T	NA	NS	Nox2	Protection by apocynin was found in WT mice but not in KO mice.	Yes
Brait [41]	2010	6–8 wk	m+f	T/P	NA	NS	Nox2	The larger infarction in male mice was dependent on both reperfusion and NOX2.	Yes (female)
De Silva [42]	2011	NA	m	T	NA	NS	Nox2	Smaller infarct volume was observed in KO mice than in WT mice.	Yes
Chen [38]	2011	12–16 wk	m	T	NA	NA	Nox2	Brain infarction was 35–44% less in KO mice compared with WT mice.	Yes
Kim [40]	2012	8–12 wk	m	P	NA	NS	Nox2	No protection by KO was found in the absence of reperfusion.	NA
MaCann [44]	2014	2–3 mo	NA	T	NA	NS	Nox2	KO showed transient nature of protection and increased revascularization.	Yes
Jackman [45]	2009	11–17 wk	m	T	NA	NS	Nox1	Cortical but not total infarct was increased in KO mice.	No
Kahles [46]	2010	NA	m + f	T/P	NA	NA	Nox1	Infarct volume was reduced by 44% after 1 h but not 2 h and pMCAO in KO mice.	Yes
Kleinschnitz [48]	2010	6–8 wk	m	T	NA	NA	Nox1	Deletion of *NOX4* but not *NOX1* or *NOX2* prevented focal ischemic injury.	No
		6–8 wk	m	T	NA	NA	Nox2		No
		6–8 wk	m + f	T/P	NA	NA	Nox4		Yes
		18–20 wk	m	T	NA	NA	Nox4		Yes

WT, wild type; BP, blood pressure; CBF, cerebral blood flow; KO, knock out; T/P MCAO, transient/permanent middle cerebral artery occlusion; m, male; f, female; NA, not available; BBB, blood brain barrier; NS, not significant.
